# Quality of life of people suffering from the diabetes type 2 – examination of the influence of various factors to prevent complications

**DOI:** 10.1186/1878-5085-5-S1-A72

**Published:** 2014-02-11

**Authors:** Halina Podbielska, Katarzyna Madziarska, Ewa Demczuk-Włodarczyk

**Affiliations:** 1 Institute of Biomedical Engineering and Instrumentation, Wroclaw University of Technology, 50-370 Wroclaw, Wybrzeze Wyspianskiego 27, Poland; 2 “Dobrzynska” Medical Center, Department of Diabetology, 50-403 Wroclaw, ul. Dobrzynska 21/23, Poland; 3 Faculty of Physiotherapy, University School of Physical Education, 51-612 Wrocław, ul. Paderewskiego 35, Poland

## Introduction

Diabetes is a chronic disease, which deteriorates the quality of life of patients and reduces the life span. In the treatment of diabetes, first behavioral methods are used, then oral anti-diabetic drugs, then, in the course of the disease progress, a combined therapy (non-insulin plus insulin) and finally, solely insulin therapy. Unfortunately, many patients are afraid of including insulin for the treatment. By delaying the proper therapy, patients expose themselves to the increased risk of complications due to the macro- and microvascular pathologies. The prevention, prediction and minimalizing of the short- and long-term complications is a main focus of health care and it is at the front of the activities of many local and global organizations (see e.g. [1,2]).

## Scientific background

Our two years long study (2011-2012) on the quality of life of the out clinic patients in Wroclaw suffered from the diabetes type 2, revealed the following: 1 quality of life was highest in patients treated with oral anti-diabetic drugs and the lowest in patients treated with insulin, only, and 2. general physical health of patients was worse than the general mental health. Moreover, we stated that the patients are more prone to develop depression than the general population. Besides of treatment method, within the factors that influence the quality of life, we found: age, disease duration, gender, obesity and presence of other diseases, including diabetes complications.

One of the frustrating for patient complication, problematic in the treatment, is development of the diabetes foot, which is very often leading to the amputation, which in turn, shorten the patients’ life. Therefore, remote monitoring of the feet condition may allow for due to time medical intervention.

## Outlook and expert recommendations

Prevention of the complication as diabetic food may be possible by assessing the conditions for the pathology development. We expect that the local temperature distribution, as well as podobarographic data differ in healthy population and diabetes patients. Multi-institutional clinical study will help to gather enough data which may be exploited for finding the predictive factors of diabetic foot development. In patients already suffering from this complication, it could be possible to react promptly in order to prevent amputation. Such an approach will allow treating patients individually, simultaneously in the most economic way.
